# Analysis of the effect of maxillary transverse deficiencies on permanent maxillary first molar rotations using 3D digital models

**DOI:** 10.1186/s12903-025-06301-x

**Published:** 2025-06-02

**Authors:** Samet Özden, Orhan Cicek

**Affiliations:** 1 Department of Orthodontics, Faculty of Dentistry, Inonu University, Malatya, 44280 Türkiye; 2 Department of Orthodontics, Faculty of Dentistry, Zonguldak Bulent Ecevit University, Zonguldak, 67600 Türkiye

**Keywords:** Maxillary transverse skeletal deficiency, Crossbite, Bilateral, Unilateral, Molar rotation angle

## Abstract

**Aim:**

The aim of this study was to evaluate the permanent maxillary first molar rotation (PMMR) angles in cases of maxillary transverse skeletal deficiency (MTSD) and to compare them with a control group.

**Materials and methods:**

In this study, which included a total of 88 patients (50 females, 38 males, with a mean age of 14.98 ± 2.14 years), consisting of 66 patients with MTSD and 22 patients in the control group, four groups were divided: Group 1 (MTSD without molar crossbite), Group 2 (MTSD with bilateral molar crossbite), Group 3 (MTSD with unilateral (right-sided) molar crossbite), and Group 4 (Control Group). Skeletal deficiencies were evaluated by measuring the interjugular, jugale right (JR), and jugale left (JL) distances on posteroanterior cephalograms (PACs), while occlusal relationships were assessed using 3-dimensional (3D) intraoral models. PMMR angles were measured using the 3D Slicer software on 3D intraoral models with the Ricketts Molar-Cusp Reference Line and the midsagittal reference plane. Statistical significance was set at *p* < 0.05.

**Results:**

The PMMR angles and JR and JL distances of Group 2 were significantly higher, while the interjugular distance was found to be the lowest (*p* < 0.05). There was no significant difference in the PMMR angles between the Control group and Group 1 (*p* > 0.05), while the JR and JL distances were significantly smaller in the Control group (*p* < 0.05). In Group 3, on the crossbite side, both the PMMR and the JR and JL distances were significantly higher than on the non-crossbite side (*p* < 0.05). A significant positive correlation was found between PMMR angles and JR and JL distances (*p* < 0.05).

**Conclusion:**

It was concluded that (i) mesiopalatal PMMRs are observed in the MTSDs with molar crossbite, (ii) molars with normal molar occlusal relationships have normal PMMR angles even in the presence of MTSDs, and (iii) early detection of MTSD enables timely interventions, preventing treatment delays and improving occlusal outcomes, particularly in developing patients, thereby optimizing long-term orthodontic results.

**Clinical relevance:**

Considering the differences in PMMR angles between MTSD patients with and without molar crossbite, these findings should be taken into account when designing expansion appliances to achieve molar derotation in these patients.

## Introduction

Angle [1], described the maxillary first molars as the ‘‘key to the occlusion’’ emphasizing their frequent alignment in a normal position and their anatomical placement within the maxilla relative to the skull base. Building on this concept, Andrews [2] later introduced his classification system, ‘‘The Six Keys to Occlusion’’, highlighting the importance of these teeth, including their rotational alignment in establashing optimal occlusal relationships. Proper positioning and rotation correction of the maxillary first molar (U1st M) within the arch are essential for minimizing anteroposterior interarch discrepancies, thereby streamlining the orthodontic treatment process [3, 4].

Maxillary transverse skeletal deficiency (MTSD) is one of the most common orthodontic anomalies, crucially compromising the integrity of the dentoalveolar structures and hindering proper facial development [5]. Due to its transverse nature, it is more frequently overlooked compared to sagittal and vertical discrepancies [6]. MTSD is typically characterized by unilateral or bilateral posterior crossbite; however, in cases with concurrent mandibular transverse deficiency, posterior crossbite may be absent. Therefore, the basic strategy for establishing a normal transverse skeletal relationship between the maxillary basal bones, crucial for achieving a successful and stable occlusion, is to initiate treatment immediately upon diagnosis [7].

In Class II malocclusions, MTSD may be obscured by the posterior positioning of the mandible, whereas in Class III malocclusions, the anterior positioning of the mandible may exacerbate the appearance of MTSD [7]. Modern approaches include a range of methods, including slow or rapid maxillary expansion using orthodontic or orthopedic forces during or prior to adolescence, as well as surgical separation of the suture palatina media in adulthood [8, 9]. It is crucial to consider that the midpalatal suture progressively fuses from adolescence into adulthood, necessitating heavier orthodontic forces to achieve effective skeletal expansion of the maxilla [9].

A balanced transverse relationship between the maxilla and mandible is essential for optimal dental function and stability, with the ideal alignment characterized by upright molars that are centrally positioned within the alveolar bone and exhibit proper intercuspation. MTSD is a common condition in patients with malocclusion, frequently presenting with crossbites, dental crowding, and wide buccal corridors. This condition is often compensated by buccal tipping of the maxillary molars and lingual inclination of the mandibular molars, which results in a pronounced curve of Wilson, altered force distribution, and potential periodontal complications [5, 10].

Unilateral maxillary transversal skeletal deficieny (UMTSD) in the intercuspal position (ICP) describes an occlusal relationship where the mandibular teeth on one side are positioned facially relative to the corresponding maxillary teeth [11]. This condition has been associated in various studies with occlusal interference, altered mandibular growth, and asymmetric muscular activity [12–14]. The treatment of UMTSD has been shown to induce favorable changes in mandibular motion [15], normalize asymmetric functional deviations, and restore balanced stomatognathic muscle activity [16]. Conversely, untreated UMTSD can lead to altered activity in certain chewing muscles during childhood and contribute to the development of craniomandibular dysfunctions in adolescence [5, 17].

Permanent first molars, which appear around the age of 6 and are critical for ensuring correct occlusion, may be adversely affected by ectopic eruption, impaction, or rotational malpositions [18]. Additionally, risks affecting tooth position on the dental arch associated with parafunctions such as thumb sucking, tongue thrusting, or mouth breathing tend to be more pronounced in genetically predisposed individuals with undesirable growth pattern [19].

Permanent maxillary first molar rotations (PMMRs), which occur due to differences in tooth size and arch size [20], are a common finding in orthodontic patients and may also occur due to premature loss of primary second molars or proximal caries. In orthodontic malocclusions, PMMRs are typically characterized by the palatal displacement of the mesiobuccal cusp, which not only reduces arch space by up to 2 mm but also contributes to the development of a Class II molar relationship [21]. Therefore, evaluation of PMMRs is critical for accurate orthodontic diagnosis and treatment planning. Additionally, the relationship between molar rotations and various orthodontic malocclusions has been investigated for many years [21]. Previous studies have reported the frequency, distribution, and prevalence of molar rotations in Class I, II, and III malocclusions [19, 22].

To our knowledge, there is no study in the literature investigating PMMRs in MTSDs. However, since the appliances used in the treatment of MTSDs are predominantly anchored to the permanent first molars, elucidating the effect of transverse deficiencies on first molar rotations is crucial for orthodontists concerning both treatment prognosis and stability. Therefore, the aim of the present study was to investigate the relationship between unilateral and bilateral MTSDs and PMMRs, and to compare them with those of the control group. The null hypothesis of the study is that there is no significant difference in PMMR angles between patients with unilateral and bilateral MTSD and the control group.

## Materials and methods

### Study design and ethical approval

This retrospective study utilized Posteroanterior Cephalograms (PACs) and 3-dimensional (3D) intraoral scans of the maxilla and mandible obtained from the clinical archives of patients divided into four groups, all of whom were referred to the Department of Orthodontics at Inonu University. Ethical approval for the study was granted by the Non-Interventional Clinical Research Ethics Committee of Inonu University (decision number 2024/6282, dated 30/07/2024). Informed consent forms were obtained from all patients and, in the case of participants under the age of 16, from their parents or legal guardians prior to the initiation of treatment; however, no additional consent was required due to the retrospective nature of the study. The study was conducted in full accordance with the ethical principles outlined in the ‘Declaration of Helsinki’.

### Sample size calculation and groups

The power analysis of the study was performed using G^*^Power software (version 3.1.9.7; Franz Faul, Kiel University, Kiel, Germany). Based on a one-way ANOVA (fixed effects, omnibus), the sample size was calculated with an effect size of f = 0.44, derived from a previous similar study by Giuntini et al. [23], a significance level (α) of 0.05, and a desired statistical power of 0.90. The analysis indicated that a minimum of 80 participants (20 per group across four groups) would be required to achieve sufficient power (actual power = 0.91, noncentrality parameter λ = 15.68, and critical F = 2.72). To enhance the reliability of the findings further, the final sample included 88 patients, with 22 patients in each group.

The inclusion criteria for the study are as follows:

- Patients aged 11 to 17 years who are eligible for the evaluation of transverse deficiencies and molar rotations.
- Complete and analyzable high quality PACs and high-quality 3D scans to ensure sufficient data for reliable analysis.
- Patients with bilateral or unilateral MTSD, or those without any crossbite or skeletal deficiency (control group).
- No missing teeth in the dental arch except for third molars.
- No history of orthodontic treatment or maxillofacial surgery to avoid potential confounding effects on molar rotation and transverse measurements.
- Patients without systemic diseases, such as connective tissue disorders, genetic syndromes, or unrelated craniofacial anomalies.
- Individuals with cleft lip/palate but no additional anomalies.
- Presence of maxillary first molars without significant restorations or prosthetic interventions.
- No extensive caries or traumatic tooth loss.
- No history of early exfoliation or extraction of the primary second molars.

Individuals who did not meet at least one of the inclusion criteria were excluded from the study, and the remaining subjects were categorized into four groups based on the transverse deficiency values obtained from PAC analyses and the occlusal relationships observed in model scanning. The groups were defined as follows:

- Group 1: MTSD group without posterior crossbite.
- Group 2: MTSD group with bilateral posterior crossbite.
- Group 3: MTSD group with unilateral (right-sided) posterior crossbite.
- Group 4: Control Group (patients without any skeletal transverse deficiency or posterior crossbite).

### Postero anterior cephalogram measurements

Posteroanterior cephalograms for all groups were obtained using an X-ray machine (Planmeca OY, Helsinki, Finland). During the acquisition of cephalometric radiographs, the head position was stabilized using cephalostats to ensure standardization, with the Frankfurt horizontal plane aligned parallel to the floor, and all radiographs were obtained while the patients were in centric occlusion. All radiographs were captured with the same device, operating at a resolution of 0.027-mm pixel size, 66 kV, 5.0 mA, and an exposure time of 18 s.

**Fig. 1:**
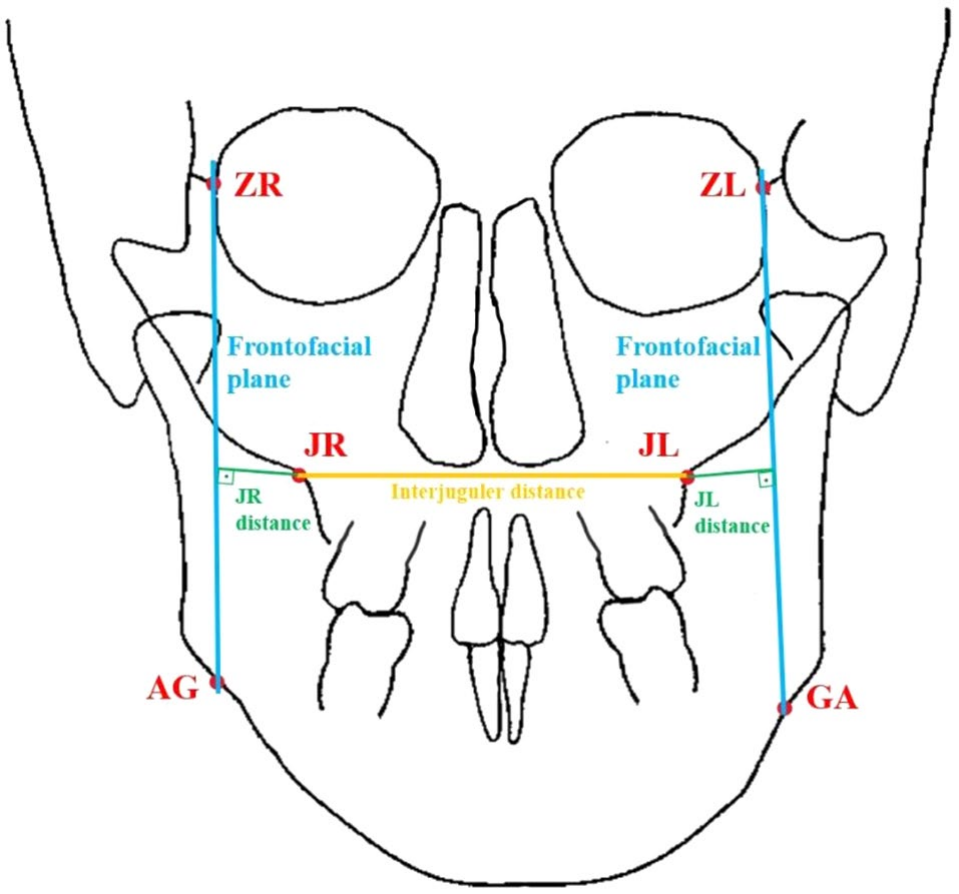
Schematic representation of the linear PAC parameters measured: Red color: PAC landmark points and abbreviations, Light blue color: Frontofacial planes, Orange color: Interjugulare distance, Green color: JR and JL distances

To identify MTSDs, two different measurements, as described by Ricketts, were performed. The first measurement was the interjugulare distance, which connects the right (JR) and left (JL) jugale points. All measurements were repeated by the same researcher (S.Ö) on 30% of the randomly selected patients at two-week intervals. The schematic representation of these linear cephalometric measurements is provided in Fig. 1. The definitions of the cephalometric landmarks and linear measurements are presented in Table 1.

**Table 1:** Cephalometric parameters and definitions

	Definition
**Cephalometric** **landmarks**	
JR point	The intersection of the right lateral contour of the maxillary alveolar process and the lower contour of the right maxillozygomatic process
JL point	The intersection of the left lateral contour of the maxillary alveolar process and the lower contour of the left maxillozygomatic process
ZR point	Point at the lateral margin of the right zygomaticofrontal suture
ZL point	Point at the lateral margin of the left zygomaticofrontal suture
AG point	The highest point of the right antegonial notch
GA point	The highest point of the left antegonial notch
**Cephalometric linear** **measurements**	
Interjugulare distance	It is distance between right and left jugal points (JR-JL)
JR distance	It is distance between from the right jugal process to the right frontofacial plane (ZR-AG)
JL distance	It is distance between from the left jugal process to the left frontofacial plane (ZL-GA)

### Maxillary first molar rotation angles measurements

The PMMR angle measurements were performed on previously scanned intraoral digital models of the patients. All scans were obtained using the same device (iTero Element 5D Dental Imaging System, Align Technology, Inc.) to ensure consistency. In the initial stage, patients were classified into two groups based on PAC analysis: those with MTSD and those without. Subsequently, intraoral scanning images were analyzed to determine the crossbite status of the patients, leading to further categorization into three groups: bilateral crossbite, unilateral crossbite, and no crossbite.

**Fig. 2:**
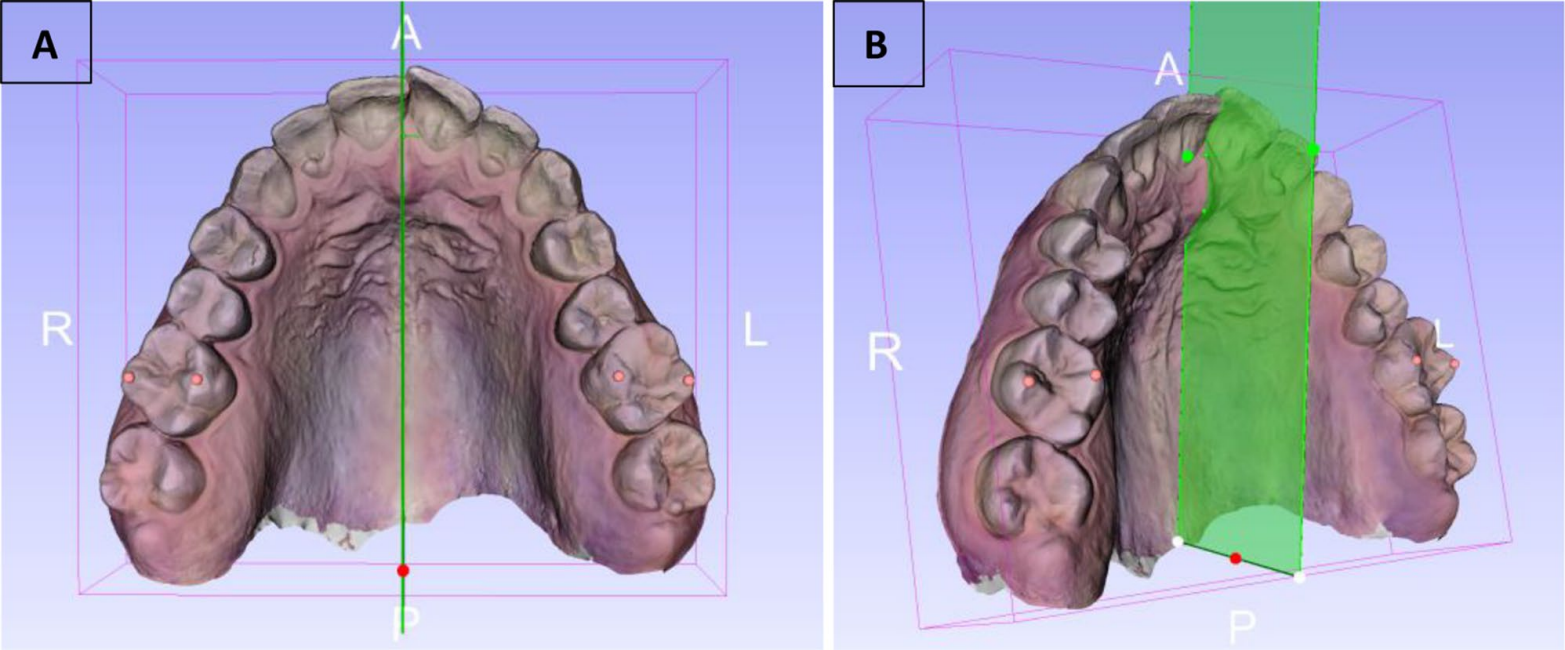
R: right, L: left, A: anterior, P: posterior. **A**) Occlusal view of the midsagittal plane drawn based on the median raphe. **B**) 3D representation of the midsagittal plane

All PMMR angle measurements were conducted using the same software (3D Slicer, version 5.6.2) to ensure consistency and accuracy across all analyses. Prior to commencing the measurements, a midsagittal reference plane was established for each maxillary scan, serving as a stable anatomical landmark. This plane was delineated based on the median raphe, which is widely recognized for its lifelong anatomical stability and reliability [24, 25] as a reference in orthodontic analyses (see Fig. 2).

**Fig. 3:**
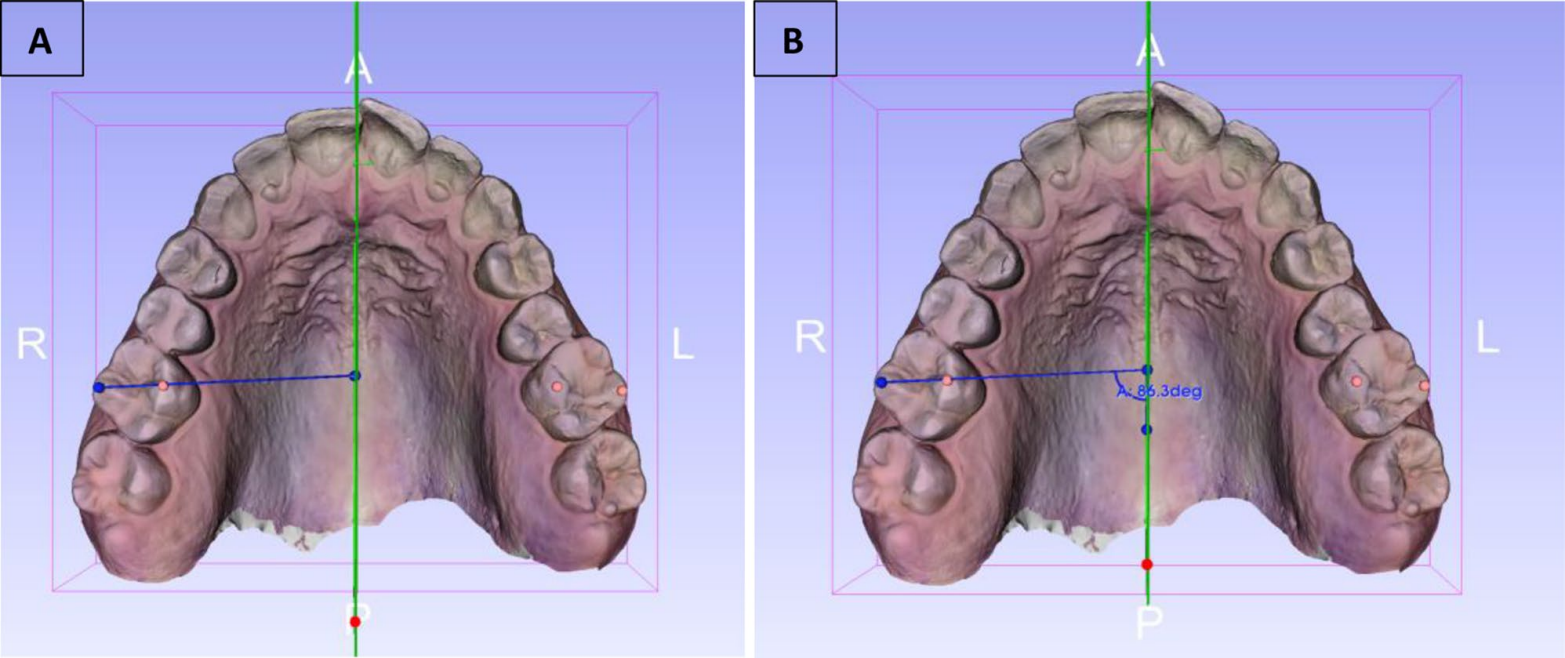
R: right, L: left, A: anterior, P: posterior. **A**) The Ricketts Molar-Cusp Reference Line connecting the disto-buccal and mesio-palatinal cusps of the right permanent maxillary first molar. **B**) Occlusal view of the molar rotation angle

**Fig. 4:**
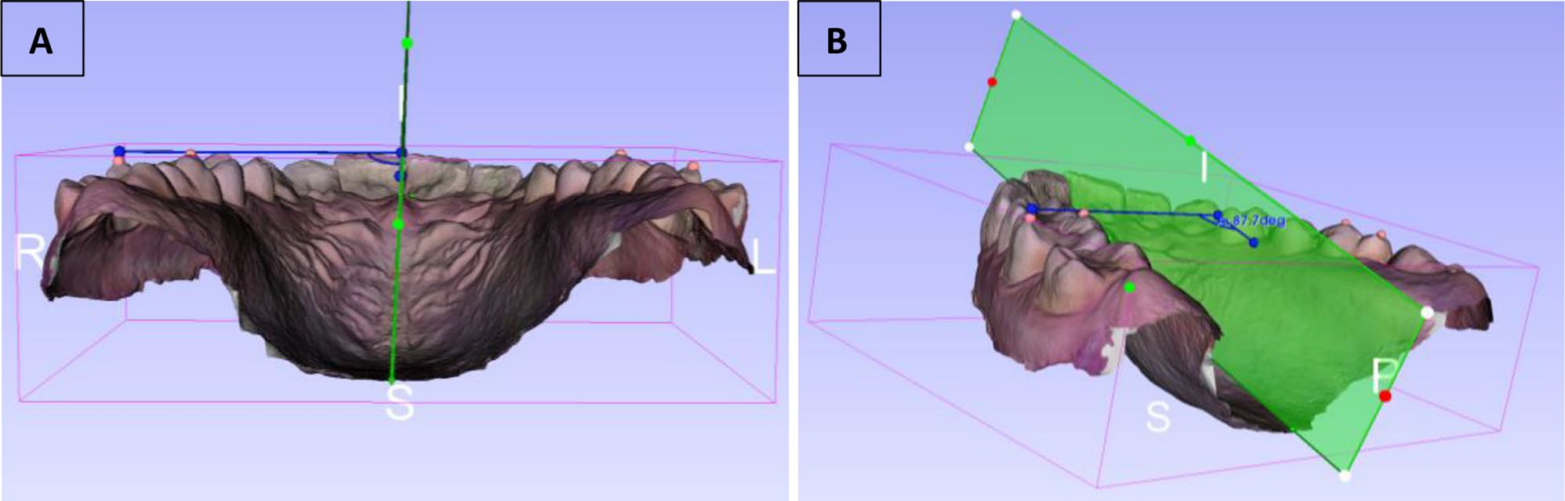
R: right, L: left, P:posterior, S: sagital plane. **A**) Orientation of the model showing the midsagittal plane (green vertical plane) intersecting the transverse plane at a 90-degree angle from a posterior perspective. **B**) Same model shown in figure A, viewed from a different perspective to demonstrate the angular measurement. The rotation angle is measured between the Ricketts Molar-Cusp Reference Line (blue line connecting the mesio-palatal and disto-buccal cusps of the maxillary first molar) and the blue line drawn on the midsagittal reference plane in 3D space

Reference points on the molar teeth were determined following Ricketts’ description of the ‘‘Ricketts Molar-Cusp Reference Line’’ [26] defined as the line connecting the mesio-palatal and disto-buccal cusps of the molars (see Fig. 3). A key feature distinguishing this study from previous research is that all angle measurements were conducted in three dimensions, allowing for a more precise and comprehensive analysis. To achieve this, a transverse plane was first established perpendicular to the midsagittal plane. This was accomplished by orienting the model such that the midsagittal plane was viewed intersecting the transverse plane at a 90-degree angle from a posterior perspective. The Ricketts Molar-Cusp Reference Line was aligned parallel to the transverse plane, ensuring accurate and consistent measurements of the angle between this line and the midsagittal reference plane in 3D space (see Fig. 4). The procedures described above were meticulously performed separately for all groups.

### Method error

To assess intraobserver reliability, repeated measurements were taken on a randomly selected 30% sample, which were repeated by the same researcher (S.Ö) with a two-week interval. Measurement error was calculated using Dahlberg’s formula:

$$\:Error\:=\:\sqrt{\frac{\sum\:{d}^{2}}{2n}}$$

where *d* represents the difference between the first and second measurement, and *n* denotes the number of subjects. Measurement errors were expressed in millimeters (mm) for linear variables and in degrees (°) for angular variables. Repeated measurements are reported to have acceptable error limits of 1 mm for linear measurements and 1.5° for angular measurements [27].

Second, Cronbach’s alpha was computed via a reliability analysis to evaluate the internal consistency of the repeated measurements. The resulting Cronbach’s alpha coefficient and its 95% confidence interval (lower and upper bounds) were interpreted to quantify the degree of agreement.

### Statistical analysis

Statistical analysis of the data, including mean and standard deviation values, was performed using SPSS Statistics version 26 (Statistical Package for Social Sciences, IBM Co., Armonk, NY, USA). Normality distributions were assessed using Kolmogorov-Smirnov and Shapiro-Wilk tests. Comparisons between groups were performed using one-way analysis of variance (ANOVA) for normally distributed data and the Kruskal-Wallis test for non-normally distributed data. Pairwise comparisons for normally distributed data, post hoc Tukey test or Tamhane’s T2 test were used based on homogeneity of variance as assessed by Levene’s statistic. Effect sizes were calculated using General Linear Model (Univariate) analysis and reported based on partial eta-squared (η²) values. The paired sample t-test was used for normally distributed data and the Wilcoxon signed-rank test for non-normally distributed data when comparing right and left measurements within groups. Pearson’s Chi-squared analysis was used for categorical variables. The relationship between molar rotation angles and JR and JL distances was assessed using Spearman’s rho correlation coefficient. The significance level was set at *p* < 0.05.

## Results

In repeated measurements, Cronbach’s alpha coefficients of at least 0.899 indicated high intraobserver reliability (*p* < 0.001). Dahlberg error values were also within acceptable limits, with values ranging from 0.48 to 1.01. These results indicate that all measurements demonstrated acceptable repeatability, as the differences between repeated measurements did not exceed the acceptable error limits of 1 mm for linear measurements and 1.5° for angular measurements (Table 2).

**Table 2:** Cronbach alpha and Dahlberg results for method error in repeated measurements

Parameters	Groups	Side	Cronbach’s alpha	95% CI lower bound	95% CI upper bound	Dahlberg error
Molar rotation angles	Group 1	Right	0.899	0.756	0.958	0.95
		Left	0.982	0.957	0.993	0.99
	Group 2	Right	0.97	0.928	0.988	1,01
		Left	0.906	0.773	0.961	0.88
	Group 3	Right	0.919	0.804	0.966	0.62
		Left	0.91	0.784	0.963	0.93
	Group 4	Right	0.958	0.899	0.983	0.52
		Left	0.928	0.826	0.97	0.48
Maxillary transverse deficiency distances	Group 1	Right	0.947	0.871	0.978	0.7
		Left	0.958	0.898	0.982	0.53
	Group 2	Right	0.908	0.779	0.962	0.68
		Left	0.901	0.761	0.959	0.95
	Group 3	Right	0.979	0.949	0.991	0.83
		Left	0.984	0.961	0.993	0.65
	Group 4	Right	0.954	0.888	0.981	0.82
		Left	0.931	0.835	0.972	0.99
Maxillary transverse width	Group 1	Interjugular distance	0.971	0.929	0.988	0.85
	Group 2	Interjugular distance	0.918	0.803	0.966	0.99
	Group 3	Interjugular distance	0.968	0.924	0.987	0.86
	Group 4	Interjugular distance	0.915	0.794	0.965	0.76

%:percentage, CI: Confidence Interval

This study included a total of 88 patients (50 female and 38 male) with a mean age of 14.98 ± 2.14 years. No significant differences were found between the groups in terms of age or gender (*p* > 0.05). The statistical analysis results for age and gender are shown in Table 3.

**Table 3:** Statistical analysis results for patient age and gender

		Group 1	Group 2	Group 3	Group 4	*p*
Age (year)	Mean ± SD	13.75 ± 2.05	15.67 ± 2.18	14.69 ± 2.02	15.8 ± 2.12	0.123 ^K^
	Median (Q1-Q3)	13 (12.3–14.7)	14.1 (11.6–16.6	14.3 (12.9–16.4)	15 (14.1–16.2)	
Gender	Female, n (%)	13 (59.1)	12 (54.5)	14 (63.6)	11 (50)	0.819 ^χ2^
	Male, n (%)	9 (40.9)	10 (45.5)	8 (36.4)	11 (50)	

Group 1: MTSD without molar crossbite, Group 2: MTSD with bilateral molar crossbite, Group 3: MTSD with unilateral (right-sided) molar crossbite, Group 4: Control Group. SD: standard deviation, %: percentage, n: sample, ^K^: Kruskall–Wallis test, ^χ2^: Chi-square test

In this study, we found significant differences in PMMR angles and JR, JL, and interjugulare distances. In Groups 1 and 4, right PMMR angles were significantly lower compared to Groups 2 and 3 (*p* < 0.05), while no significant differences were observed between Groups 1 and 4 (*p* > 0.05). The right PMMR angle was found to be significantly highest in Group 2 (*p* < 0.05). The right PMMR angle in Group 3 was significantly higher than in Groups 1 and 4, yet significantly lower than in Group 2 (*p* < 0.05). The left PMMR angle was found to be significantly highest in Group 2 (*p* < 0.05), while no significant differences were observed between the left PMMR angles of Groups 1, 3, and 4.

In the within-group comparisons, the right PMMR angle in Group 3 was found to be statistically significantly higher than the left PMMR angle (*p* < 0.05), whereas no significant differences were observed between the right and left PMMR angles in Groups 1, 2, and 4 (*p* > 0.05).

The JR distance in Group 1 was found to be significantly lower than in Groups 2 and 3, while it was higher than in Group 4 (*p* < 0.05). However, the JR distances in Groups 2 and 3 were significantly higher than those in Groups 1 and 4, while no significant differences were observed between Groups 2 and 3. The JR distance in Group 4 was found to be significantly the lowest (*p* < 0.05). In terms of JL distance, Group 1 was significantly lower than Group 2, while no significant differences were found between Group 1 and Groups 3 and 4. The JL distance was found to be significantly the highest in Group 2 (*p* < 0.05). The JL distance in Group 3 was significantly lower than in Group 2 and significantly higher than in Group 4, while no significant differences were observed between Group 3 and Group 1. The JL distance in Group 4 was significantly lower than in Groups 2 and 3 (*p* < 0.05), while no significant differences were observed between Group 4 and Group 1 (*p* > 0.05).

In the within-group comparisons, the JR distance in Group 3 was significantly greater than the JL distance (*p* < 0.05), while no significant differences were observed between the JR and JL distances in the other groups (*p* > 0.05).

For Group 1, the first quartile (Q1), median (Q2), and third quartile (Q3) values of the JR distance were 10.71, 11.50, and 12.30, respectively. For this group, the Q1, median (Q2), and Q3 values of the JL distance were 9.94, 10.62, and 12.55, respectively. For Group 3, the JR distance’s Q1, median (Q2), and Q3 values were 12.47, 13.23, and 14.27, respectively, while the JL distance’s were 10.42, 11.26, and 12.76.

The interjugulare distances measured for the maxillary transverse width were found to be significantly higher in Group 4 compared to Groups 1 and 2 (*p* < 0.05), while no significant differences were observed between the interjugulare distances of Groups 1, 2, and 3 (*p* > 0.05).

The results of the statistical analysis for within and between group comparisons are presented in Table 4.

**Table 4:** Comparison of molar rotation angles and transverse measurements between and within groups with effect sizes (Partial η²)

Variables		Group 1^a^ (Mean ± SD)	Group 2^b^ (Mean ± SD)	Group 3^c^ (Mean ± SD)	Group 4^d^ (Mean ± SD)	Effect size (Partial η²)	*p*
Molar rotation angles	Right angle	76.35 ± 4.45^b, c^	84.65 ± 4.91^a, c,d^	80.46 ± 5.18^a, b,d^	73.65 ± 4.43^b, c^	0.446	**< 0.001** ^*,A^
	Left angle	76.77 ± 5.14^b^	83.17 ± 4.94^a, c,d^	75.08 ± 4.79^b^	73.87 ± 4.79^b^	0.357	**< 0.001** ^*,A^
	Intra-group difference *p*	0.509 ^P^	0.201 ^P^	**< 0.001** ^*,P^	0.725 ^P^		
Maxillary transverse deficiency distances	JR distance	11.48 ± 1.25^b, c,d^	13.52 ± 1.20^a, d^	13.56 ± 1.65^a.d^	10.35 ± 1.07^a, b,c^	0.533	**< 0.001** ^*,A^
	JL distance	11.13 ± 1.54^b^	12.96 ± 1.07^a.c.d^	11.53 ± 1.67^b, d^	10.10 ± 0.95^b, c^	0.377	**< 0.001** ^*,A^
	Intra-group difference *p*	0.559 ^W^	0.112 ^P^	**< 0.001** ^*,W^	0.239 ^P^		
Maxillary transverse width	Interjugular distance	60.33 ± 3.19^d^	58.27 ± 5.19^d^	60.54 ± 3.56	62.77 ± 2.22^a, b^	0.163	**0.002** ^*,A^

Group 1: MTSD without molar crossbite, Group 2: MTSD with bilateral molar crossbite, Group 3: MTSD with unilateral (right-sided) molar crossbite, Group 4: Control Group. ^A^: one-way ANOVA (analysis of variance) test, ^P^: Paired t test, ^W^: Wilcoxon signed ranks test. ^*^: *p* < 0.05. JR: Jugale right, JL: Jugale left. SD: standard deviation. η²: eta squared

^a^: Difference with Group 1 in the same row *p* < 0.05, ^b^: Difference with Group 2 in the same row *p* < 0.05, ^c^: Difference with Group 3 in the same row *p* < 0.05, ^d^: Difference with Group 4 in the same row *p* < 0.05

The PMMR angles and JR and JL distances of Group 2 were significantly higher, while the interjugulare distance was found to be the lowest (*p* < 0.05). There was no significant difference in the PMMR angles between the Control group and Group 1 (*p* > 0.05), while the JR and JL distances were significantly smaller in the Control group (*p* < 0.05). In Group 3, on the crossbite side, both the PMMR angle and the JR and JL distances were significantly higher than on the non-crossbite side (*p* < 0.05). A significant positive correlation was found between PMMR angles and JR and JL distances (*p* < 0.05).

**Fig. 5:**
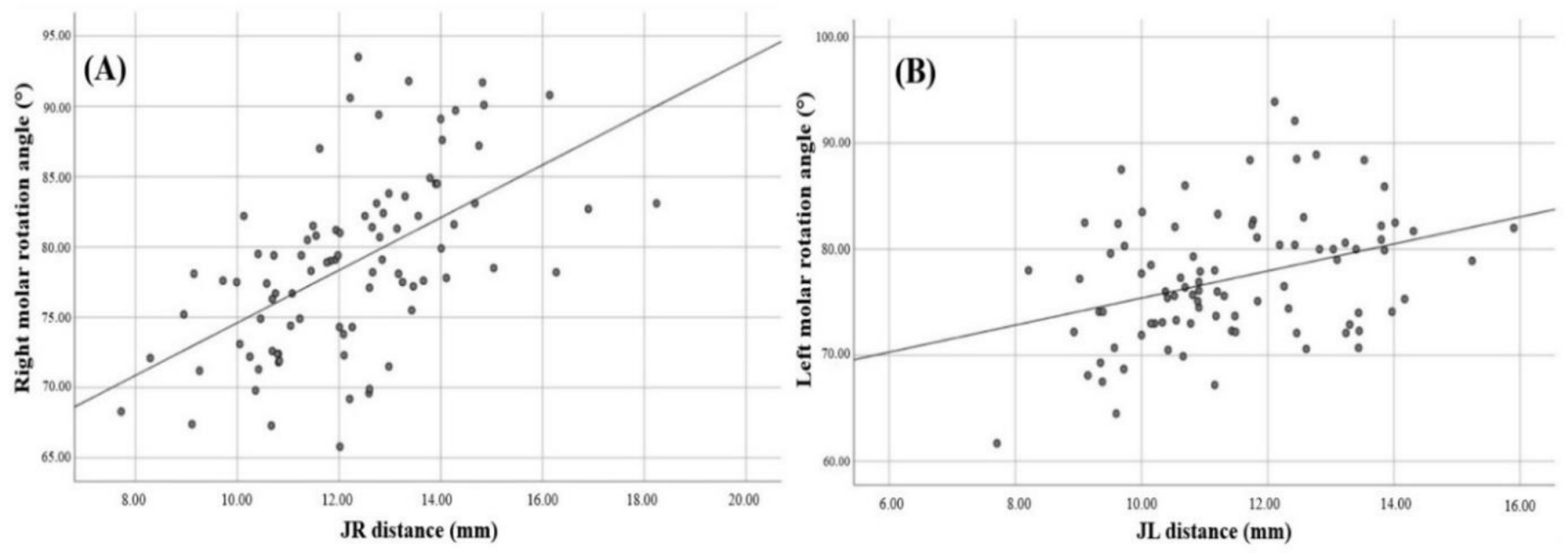
Scatter plots illustrating the correlation between molar rotation angles and JR and JL distances: (**A**) the correlation between the right molar rotation angle and JR distance, (**B**) the correlation between the left molar rotation angle and JL distance

As the JR distance, which causes MTSD on the right side, increased, a significant increase in the right PMMR angle was also observed (*p* < 0.05). Similarly, on the left side, the left PMMR angle was found to increase as the JL distance, which causes the MTSD, increased (*p* < 0.05). Accordingly, a statistically significant positive correlation was found between the right PMMR angle and JR distance, as well as between the left PMMR angle and JL distance (*p* < 0.05). The results of the correlation analysis are presented in Table 5. In addition, the scatter plots for the statistically significant positive correlations between right PMMR angle and JR distance and between left PMMR angle and JL distance are shown in Fig. 5.

**Table 5:** Correlation analysis results between PMMR angles and JR and JL distances

		Right PMMR angle	JR distance	Left PMMR angle	JL distance
Right PMMR angle	Spearman rho	1	0.601	0.681	0.450
	*p*		**< 0.001** ^*^	**< 0.001** ^*^	**< 0.001** ^*^
JR distance	Spearman rho		1	0.338	0.576
	*p*			**0.001** ^*^	**< 0.001** ^*^
Left PMMR angle	Spearman rho			1	0.328
	*p*				**0.002** ^*^
JL distance	Spearman rho				1

^*^: *p* < 0.05. PMMR: permanent maxillary first molar rotation, JR: Jugale right, JL: Jugale left

## Discussion

Our study’s findings reveal that varying degrees of PMMR angles are observed among individuals with different types of MTSD, highlighting the nuanced relationship between maxillary morphology and dental alignment. The significant correlation observed between the MTSD distances and PMMR angles across the groups indicates that as the severity of transverse deficiency increases, PMMR angles are correspondingly affected. This finding underscores the close relationship between transverse maxillary deficiency and rotational compensations in the molars, suggesting that greater skeletal deficiencies may exacerbate rotational misalignments. Therefore, the null hypothesis of the study was rejected.

The literature includes studies investigating maxillary first molar rotations in sagittal (Class I, Class II, and Class III) [28–32] and vertical (hypodivergent and hyperdivergent) [33] malocclusions. These studies have reported a higher prevalence of mesiopalatal molar rotation in individuals with skeletal Class II malocclusions, with the severity of Class II malocclusion correlating with an increased incidence of this rotation [28–32]. Additionally, it has been noted that individuals with a vertical growth pattern, characterized by an increased SN/MP angle, exhibit a greater tendency for mesiopalatal rotation of maxillary first molars [33]. However, no studies have been identified in the literature that investigate the relationship between the presence of dental or skeletal transverse deficiency and maxillary first molar rotations.

Maxillary transverse skeletal deficiency should be promptly treated upon diagnosis to restore a proper transverse skeletal relationship between the basal bones, which is essential for attaining a stable and functional occlusion [7]. Indeed, sagittal malocclusions spontaneously corrected after correction of the transversal deficiency, according to McNamara [5]. However, the adaptation of especially banded rapid maxillary expansion (RME) appliances can be particularly challenging in the presence of severely rotated maxillary first molars, necessitating the correction of these rotations prior to initiating expansion treatment. This not only delays the treatment process but also allows further fusion of the maxillary sutures during this period, thereby diminishing the skeletal effectiveness achievable with RME. In this context, the present study aims to investigate the potential association between bilateral and unilateral skeletal and dental maxillary transverse deficiency and the rotational alignment of the maxillary first molars. The findings are anticipated to contribute to earlier and more accurate diagnoses, particularly in MTSD cases requiring timely intervention during early developmental stages, thereby preventing potential delays in treatment and improving overall clinical outcomes.

Various methods have been employed to diagnose maxillary transverse deficiency, particularly to distinguish between the dental and skeletal characteristics of malocclusion. These methods include dental cast analysis [5, 34, 35], analyses conducted on PACs [36, 37], Pont’s index [38] and measurements obtained from CBCT scans [39–41].

For many years, the assessment of maxillary transverse dimensions obtained from PACs has served as a standard guide for evaluating the need for maxillary expansion procedures. Ricketts developed a transverse diagnostic method based on PAC analyses and established a set of age-specific normative values to guide clinical assessments [42, 43]. Tai et al. [44] conducted maxillary transverse width measurements using both CBCT and PAC’s and reported no significant difference between the two imaging methods in terms of interjugulare distance measurements. In our study, PACs were utilized for measurements due to their advantages, including low radiation dose and the ability to simultaneously and efficiently assess both the dental and skeletal characteristics of maxillary deficiency using a single method.

Three measurements described in Ricketts’ PAC analysis were used in this study for assess the MTSD: interjugulare distance (JR-JL), JR distance (ZR-AG to JR) and JL distance (ZL-GA to JL) [43]. The JR-JL distance was used to assess whether the maxilla exhibits transverse deficiency relative to the general population’s normative values. Meanwhile, JR distance and JL distance were utilized to evaluate the symmetry of the maxilla in relation to the total face as well as the differences between the right and left sides, which are particularly crucial for accurately diagnosing unilateral transverse deficiencies.

Rickets [26] suggested that in maxillary models, the line connecting the disto-buccal and mesio-palatal cusp crests of the first molars should pass through the distal 1/3 of the opposite canine. In contrast, Cetlin and Ten Hoeve [45] reported that molars are well positioned if their buccal surfaces are parallel to each other. There are various methods to evaluate PMMRs. Vigano et al. [28] performed measurements on photocopies by determining the angles formed at the intersection of lines passing through the mesio-palatal and disto-buccal cusps of each molar (Ricketts Molar-Cusp Reference Line) with a straight line traced along the palatine raphe, using a 0.5 mm graphite pencil for precise reference tracing. Naushad et al. [21] also performed rotation measurements on photographs derived from dental models. Zingaretti et al. [29] conducted rotation measurements using two-dimensional (2D) images obtained by scanning dental models, which were analyzed through specialized software. Alexander et al. [31] utilized a leveling bubble to standardize digitally photographed models, measuring molar rotation along with parameters such as the angle of Friel, Ricketts’ E-line, Henrys’ angle, and the premolar angle using a digital protractor. All of the aforementioned studies, while employing various methods, were limited to 2D measurements, which inherently restrict the ability to accurately capture the complete rotational positions and spatial relationships of the teeth.

Such limitations become particularly significant when assessing complex dental and skeletal malocclusions, as 2D methods fail to provide a comprehensive representation of the anatomical structures involved. In contrast, our study utilizes 3D measurements of rotational angles, offering a more precise and detailed analysis that enhances the accuracy of spatial evaluations and improves treatment planning. By integrating 3D measurement techniques, this study not only overcomes the constraints of traditional methods but also contributes a novel perspective to the literature, advancing the understanding of the intricate relationship between maxillary deficiency and molar rotations. In measurements conducted using the Ricketts Molar-Cusp Reference Line, the median raphe was chosen as the vertical reference line to ensure standardization. This approach provides a consistent and anatomically stable landmark, allowing for accurate and reproducible assessments of molar rotations [24, 46].

When all groups were analyzed, the highest PMMR angle values were observed in patients with bilateral skeletal MTSD accompanied by bilateral crossbite (Group 2). This finding may be attributed to the increased compensatory rotation of molars in response to the transverse imbalance and occlusal discrepancies characteristic of bilateral crossbite cases. The dual presence of skeletal deficiency and crossbite likely exacerbates the rotational misalignment as the dentition adapts to the restricted maxillary arch width and altered functional occlusion.

No significant difference in PMMR angles was found between the MTSD without crossbite group (Group 1) and the control group (Group 4), suggesting that MTSD alone does not significantly affect PMMR in the absence of occlusal discrepancies such as those caused by a crossbite. The absence of crossbite likely allows for more balanced occlusal forces and alignment, reducing the need for compensatory molar rotation. In contrast, individuals with an unilateral crossbite (Group 3) had significantly greater PMMR angles on the affected side (right side) compared to the non-affected side (left side). This discrepancy likely reflects the localized transverse imbalance and asymmetrical occlusal forces exerted on the crossbite side, which increase compensatory molar rotation. The altered occlusal dynamics and restricted arch space on the crossbite side may further contribute to this rotational misalignment, highlighting the critical role of occlusal and skeletal symmetry in maintaining proper molar alignment.

An intriguing finding reported by Naushad et al. [21], Maharjan et al. [32], and Shaheen et al. [33] is the significant difference in molar rotation angles between the right and left sides. However, the underlying cause of this asymmetry was not explored in detail within their studies. We attribute that this discrepancy in molar rotation angles may be due to variations in the transverse malocclusions present in the patients, as different types of transverse discrepancies could lead to uneven occlusal forces and asymmetrical molar rotations.

Recent advances in artificial intelligence (AI) have significantly contributed to various aspects of orthodontic diagnosis and treatment planning. In particular, the integration of AI into 3D dental imaging and digital model analysis has enhanced diagnostic precision and efficiency, particularly in malocclusion assessment and cephalometric evaluations [47, 48]. Although AI was not directly employed in the present study, the use of 3D software in measuring molar rotation angles aligns with the ongoing digital transformation in orthodontics. Future studies may further benefit from incorporating AI-based automated detection and classification tools for skeletal discrepancies.

The limitations of this study primarily arise from the use of 2D PACs for transverse measurements, which cannot fully represent the 3D relationships and depth of anatomical structures. However, the rotational measurements of the maxillary first molars were performed on digital models using 3D software. Moreover, these measurements were performed based on standardized reference points such as the median raphe, and the accuracy of the measurements was found to be reliable in repeated assessments. Additionally, other limitations include the lack of a comparison of the compensatory rotational angles of the mandibular first molars and the inclusion of only right-sided unilateral crossbite cases due to the asymmetrical distribution of available data. Despite these limitations, this study’s results provide orthodontists with valuable clinical insights into molar crossbite and occlusion when treating MTSD cases. Our findings contribute to the understanding of the nature of molar rotations in cases of maxillary transverse deficiency, thereby providing insights for planning molar derotations during appliance design prior to expansion and in fixed treatment prescriptions following expansion.

## Conclusions

The null hypothesis of the study was rejected. The following conclusions were reached based on the differences in PMMR angles in MTSD patients with and without molar crossbite:

- In bilateral crossbite cases, PMMR angles and JR–JL transverse deficiency measurements were found to be the most pronounced, whereas no such increase was observed in non-crossbite transverse deficiency cases.
- These findings indicate that the mesiopalatal rotations frequently seen in crossbite cases may lead to overcorrection and non-occlusion when using rigid expansion appliances. Since a certain degree of mesiobuccal rotation is typically achieved during fixed orthodontic treatment due to bracket prescription mechanics, these initial rotations should be carefully evaluated during treatment planning.
- Significant positive correlations between PMMR angles and JR/JL distances should inform the design of appliances for cases of skeletal MTSD, with or without a crossbite. Before placing TPA after expansion, the first molars can be derotated by applying the appropriate rotational torque.

## Data Availability

The datasets used and/or analysed during the current study are available from the corresponding author on reasonable request.

## Abbreviations

MTSD: Maxillary Transverse Skeletal Deficiency
UMTSD: Unilateral Maxillary Transversal Skeletal Deficieny
PMMR: Permanent Maxillary First Molar Rotation
PAC: Posteroanterior Cephalogram
3D: Three Dimensional
kv: Kilovolt
mA: Miliamper
CBCT: Cone Beam Computed Tomography
RME: Rapid Maxillary Expansion
